# Capitals diminished, denied, mustered and deployed. A qualitative longitudinal study of women's four year trajectories after acute health crisis, Burkina Faso

**DOI:** 10.1016/j.socscimed.2012.09.025

**Published:** 2012-12

**Authors:** Susan F. Murray, Mélanie S. Akoum, Katerini T. Storeng

**Affiliations:** a King's College London, James Clerk Maxwell Building, 57 Waterloo Road, London SE1 8WA, UK; b Agence de Formation, de Recherche et d' Expertise en Santé pour l'Afrique (AFRICSanté), 01 BP 298 Bobo-Dioulasso 01, Burkina Faso; c London School of Hygiene & Tropical Medicine, Keppel Street, London WC1E 7HT, UK; d Centre for Development and the Environment, University of Oslo, Postboks 1116, Blindern, 0317 Oslo, Norway

**Keywords:** Burkina Faso, Qualitative longitudinal research, Maternal morbidity, Social capital, Bodily capital, Resilience, Trajectories, Costs of healthcare

## Abstract

Accumulating evidence indicates that health crises can play a key role in precipitating or exacerbating poverty. For women of reproductive age in low-income countries, the complications of pregnancy are a common cause of acute health crisis, yet investigation of longer-term dynamics set in motion by such events, and their interactions with other aspects of social life, is rare. This article presents findings from longitudinal qualitative research conducted in Burkina Faso over 2004–2010. Guided by an analytic focus on patterns of continuity and change, and drawing on recent discussions on the notion of ‘resilience’, and the concepts of ‘social capital’ and ‘bodily capital’, we explore the trajectories of 16 women in the aftermath of costly acute healthcare episodes. The synthesis of case studies shows that, in conditions of structural inequity and great insecurity, an individual's social capital ebbs and flow over time, resulting in a trajectory of multiple adaptations. Women's capacity to harness or exploit bodily capital in its various forms (beauty, youthfulness, physical strength, fertility) to some extent determines their ability to confront and overcome adversities. With this, they are able to further mobilise social capital without incurring excessive debt, or to access and accumulate significant new social capital. Temporary self-displacement, often to the parental home, is also used as a weapon of negotiation in intra-household conflict and to remind others of the value of one's productive and domestic labour. Conversely, diminished bodily capital due to the physiological impact of an obstetric event or its complications can lead to reduced opportunities, and to further disadvantage.

## Highlights

► Qualitative longitudinal research in Burkina Faso followed women for four years after a costly obstetric complication. ► Women's social capital ebbs and flows over time, resulting in a trajectory of multiple adaptations. ► Customary obligation and reciprocity provide some protection but also bring indebtedness and loss of status. ► Mobility is a feature of social life, and temporary self-displacement is used to resolve problems and secure advantage. ► Women's ability to harness bodily capital is key in overcoming adversity and in accessing new social capital.

## Introduction

Health crises are known to have physical, financial and social effects that are particularly challenging for poor households, and these effects exacerbate each other in contexts where services are not free at the point of use. Research into causes of impoverishment in developing countries suggests that most household descents into destitution are incremental, ‘pushed along by successive everyday events’ (Krishna, 2010:17) and highlights that health crises play a major role in catalysing or increasing vulnerability to further loss. Costly healthcare causes sale of essential assets and household debt (Russell & Gilson, 2006; Su, Kouyate, & Flessa, 2006; Van Damme, Van Leemput, Por, Hardeman, & Meessen, 2004). Delays in essential care-seeking or default from follow-up can result in morbidities and disabilities that further damage capacity to generate livelihoods (Mugisha, Kouyate, Gbangou, & Sauerborn, 2002; Sauerborn, Adams, & Hien, 1996; Storeng, Akoum, & Murray, 2012).

For women of reproductive age in low-income countries, the complications of pregnancy are a common cause of major health crisis (Ribeiro, Jacobsen, Mathers, & Garcia-Moreno, 2008), yet investigation of longer-term dynamics set in motion by such events, and their interactions with other aspects of social life, is rare. In this article, we present longitudinal qualitative research on 16 women's trajectories in the four years after acute pregnancy-related health crises in Burkina Faso. We employ an analytical strategy examining continuity and change, and the concepts of social and bodily capital, to understand how essential social resources are diminished, denied, mustered and deployed within and across poor households.

## Resilience, social and bodily capital

‘Resilience’ and ‘social capital’ are concepts widely used in the health and social development literature. Each has been the subject of debate and diversity of use and meaning (see for example, Bebbington, 2007; Krishna, 2002; Manyene, 2006; Obrist, Pfeiffer, & Henley, 2010; Portes, 1998). Individuals, communities or nations can be said to have a degree of resilience, which can be defined in terms of their primary survival values or assets—life, livelihoods and culture (Manyene, 2006). As Manyene's recent review highlights, the essence of an individual's ‘resilience’ is typically seen as centring on quick recovery from shock, illness or hardship. A resilient person is often thought to be one who ‘bounces back’. However, as the anthropologist Obrist (2010:282) emphasises, it is more useful to understand ‘resilience’ as a process rather than a quality, and to describe the ‘trajectories of adaptation’ (Henley, 2010:297) that occur after crises. Through the synthesis of case studies presented in this paper, we aim to emphasise the value of examining adaptive trajectories over several years by taking the detailed ‘longer look’.

Social capital is often seen as a key contributor to resilience. In the context of Western societies, early sociologists emphasised the positive consequences of group life (Portes, 1998). Later, Bourdieu, drawing attention to various resources that determine human agency, used ‘social capital,’ to refer to material and non-material resources that individuals can mobilise by virtue of many different kinds of social relationships (Bebbington, 2007; Bourdieu, 1984). He contrasted ‘social capital’ with ‘cultural capital’ – a person's formal education and other different sets of cultural competencies acquired through socialisation – and ‘economic capital’ – money and material assets. Command of capitals enables one to exercise and resist domination in social relations, to maintain a position in the status hierarchy of society. The unequal distribution of structurally based resources (capitals) is thus part of the fundamental system of inequality in a given society, and is both the result and a key mechanism of the social reproduction of power and privilege (Abel & Frohlich, 2012; Bourdieu, 1984). The different forms of capital are interrelated; it is their interactions that permit social inequalities to endure over time.

Whether used to depict an individual or a collective asset, the general consensus is that “social capital stands for the ability of actors to secure benefits by virtue of membership in social networks or other social structures” (Portes, 1998:6). Putnam, for example, saw social capital as an inherent property of communities and developed measures to assess the “features of social organisation such as networks, norms and social trust that facilitate coordination and cooperation for mutual benefit” (Putnam, 1995:67).

Bourdieu's (1986) interest was to describe the mechanisms by which the privileged retain and protect their advantage. Subsequent writers have used the concept of social capital to explore social relations in poor communities (e.g. Narayan & Pritchett, 1997), and to develop policy strategies of ‘poverty alleviation’ through the strengthening of associational life. However, this idea that social capital can be readily created, used, or substituted for other missing assets, and thereby overcome poverty, has also been strongly challenged. Cleaver's (2005) ethnography in Tanzania, for example, demonstrates that the poorest experience clusters of interlocking disadvantage and that social relationships, collective action, and local institutions may structurally reproduce the exclusion of the poorest. Moreover, as Bebbington (2007:157) and others have reiterated, Bourdieu's conceptualisation is of social capital that ‘includes and excludes at one and the same time’.

From this perspective, social capital cannot be separated from the distribution of resources and power relationships of which it is a part and which it serves to reproduce. Nor can it be separated from norms and ‘common sense’ assumptions that help naturalise the forms taken by social capital, and that are, in turn, reproduced by them. Feminist analyses have emphasised that social capital – and its downside – take gender-differentiated forms (van Dijk, 2011; Mayoux, 2001; Silvey & Elmhurst, 2003). For example, Maclean's (2010) study of micro-financing initiatives in Bolivia, demonstrates that to understand social capital simply as a ‘public good’ overlooks the restrictions imposed by relationships, tradition and norms, and the gendered contradictions of social capital. Maclean's work indicates explanatory value in treating social capital as individual assets (Maclean, 2010; Portes, 1998). In this article we, too, privilege the perspectives of individual women, rather than those of ‘households’, in order to illuminate the interior of household relations as well as the roles played by kinship and community networks in the momentum of individual women's lives.

We also employ the notion of ‘bodily capital’, a term not widely applied in literature on health and illness or on social development. Bourdieu (1984) alludes briefly to bodily capital, both inherited and acquired, as also constituting a resource that gives an individual leverage in social struggles. Wacquant's (1995, 2004) ethnography of professional boxers in the USA took this forward, examining how fighters conceived or, cared for and rationalised the use of their body as a form of capital embedded in a social setting that puts a high premium on physical prowess. Here, we use the concept in order to emphasise that the human body is often poor people's main productive asset, ‘which unlike most assets can flip or slide from being an asset to being a liability’ (Krishna, 2010:87). Recognition of the role of bodily capital, and also of its gender-specific nature, would seem to be important in societies like that of Burkina Faso where physical labour, reproductive capacity and other valued physical attributes have high social value.

## The setting

The study was carried out in Burkina Faso, a former French colony in West Africa that is one of the world's poorest countries (UNDP, 2009). In the Western regions where this data collection took place, many households rely on some combination of subsistence agriculture, petty trade, and seasonal wage labour for the state-owned cotton company. Migrant labour often contributes to household revenue, but has reduced with political instability in neighbouring Cote d’Ivoire. There is a high female labour participation rate, 82% in 2008 (ILO, 2009), although women's labour is largely confined to the informal sector of the economy.

About half the population belonging to the Mossi, one of some sixty ethnic groups. About half of the population is Muslim. Households can be extensive, typically mutigenerational and may include monogamous or polygynous marriages. Fertility rates are high (5.9 for 2005–2010; UNDP, 2009). One in fifty-five women are estimated to die from a pregnancy-related cause (World Health Organization, 2012). This reflects endemic and structural poverty, but also a weak health system unable to deal adequately with pregnancy-related complications (Hounton et al., 2005). During the study period, maternal healthcare was financed almost exclusively by user fees. Later, government subsidies were introduced to help protect households from catastrophic healthcare expenditure, although effectiveness of this policy is not yet clearly established (Ridde, Richard, Bicaba, Queuille, & Conombo, 2011).

## Study design

There are a number of ways to collect data concerning processes during, and ‘trajectories of adaptation’ after, health crises. Obrist et al. (2010) use cross-sectional qualitative methods to study how people in Kilombero Valley, Tanzania mobilised, combined and transformed capitals on the household and community levels to obtain malaria treatment. Krishna, 2005 used community focus groups and one-off individual in-depth interviews with senior male and female household members and household data to identify common pathways into and out of poverty. Russell and Gilson (2006) used in-depth longitudinal study of 16 case study households over 8 months for detailed investigation of illness costs and livelihood impacts over time in Sri Lanka.

We argue that prospective qualitative longitudinal research (QLR) is particularly appropriate to a trajectory-focused exploration of health crises and of the dynamics of social capital. In particular, we wish to suggest, an analytical focus on social change and continuity in the lives of individuals illuminates critical aspects of the *process* of resilience or adaptation. Prospective qualitative longitudinal research (QLR) aims to ‘walk alongside’ individuals or groups over time and contributes to a strand of social research that “follows the temporal rhythms of lived lives” (Mcleod & Thomson, 2009:60). QLR thus lends itself to the study of transitions, of how pathways are constructed, and the impact of key events and changing circumstances (Elliot, Holland, & Thomson, 2008; Holland, Thomson, & Henderson, 2006).

The ethics committees of the London School of Hygiene & Tropical Medicine and Ministry of Health, Burkina Faso approved the study. Data collection was conducted in two distinct waves. In both waves, our in-depth investigation was nested within a prospective epidemiological cohort study investigating health, social and economic consequences of obstetric emergencies (Filippi et al., 2007). That study focused on ‘near-miss’ obstetric events - complications that would likely have killed the woman had she not received timely medical care. In the first wave, qualitative fieldwork with 82 women recruited in two major cities and several small towns in Burkina Faso, took place over one year (Storeng et al., 2008). Initial work examined the immediate aftermath of these critical events, the immediate financial costs of emergency hospitalisation to poor households (Storeng et al., 2008) and the ensuing loss experienced by women through the combined disruptions of bodily integrity, of the household economy, and of social identity and social stability (Storeng, Murray, Akoum, Ouattara, & Filippi, 2010).

The QLR sample used in this article comprises sixteen of the twenty two women who were recruited from two hospitals in the western part of the country for the epidemiological study in 2004/2005, followed up for a year, and re-contacted for the second wave three to four years later. They had all survived near-miss events after treatment in either a referral-level hospital or a regional hospital, with pregnancy outcomes ranging from termination to stillbirth to live-birth. At their entry into the epidemiological study they were approached regarding participation in additional confidential in-depth qualitative interviews about their lives and about their recent healthcare experiences. It was made clear to them that there was no obligation to take part in this element. All those approached agreed to participate in the audio-recorded in-depth interviews, and agreed to the possibility of return visits by the researchers to request follow-up interviews. The researcher then contacted or visited the women to arrange interviews at the time and place of their choice (usually their home). On each occasion the interviewee was given the opportunity not to continue. One woman did decline to participate in the second wave follow-up. Four were now living elsewhere and could not be reached, and one had died from an unsafe abortion in the intervening period.

Ages at recruitment ranged from 14 to 40 years. The women had from zero to ten children. Some had experienced this ‘near miss’ in their first pregnancy, while others had a series of reproductive disruptions. The group encompassed Muslim, animist and Christian, women with schooling and without, single women and women in polygynous and monogamous marriages, women living in towns with ready geographical access to healthcare and women living in isolated villages with dirt roads, whose ‘near miss’ classification would readily have converted into a ‘maternal death’ if it had occurred in the rainy season.

Interviews were conducted by MA and KS in the first wave, and by MA in the second, in the woman's primary language (usually Mooré, Dioula or French). The material is wide ranging and encompasses women's narratives about their sense of self, their attitudes to their past and their future, experiences with work, spouses, parents, children, households, pregnancy, illness and healthcare. When possible, we also spoke with or interviewed women's husbands or other relatives. The interviews were tape-recorded and MA simultaneously translated and transcribed the interviews in French. This reduces the potential risk of loss of information through a multi-stage translation process but removes the option of using back translation as a quality check. The use of the same interviewer in the two waves was predicated on the likely benefits of continuity of researcher and researched for incremental development of data (Mcleod & Thomson, 2009).

## The analysis process

The co-authors independently read and conducted preliminary analysis on the interview transcripts and fieldnotes. French transcripts of the follow-up interviews were also translated into English and we worked across these two languages during the analysis. This allowed each team member to immerse themselves in the data using transcripts in the language in which they were most fluent. During two residential analysis workshops lasting a total of three weeks and conducted primarily in French, we then discussed interpretations of data and the emerging themes that we had each identified. Together, we initiated a more structured thematic analysis using a Longitudinal Coding Matrix. We adapted Saldana's (2009) matrix template to log summary observations drawn from the large amounts of data for each study participant. In this template, selected change processes are attributed to qualitative data collected and compared across time, using seven descriptive categories:

• What increases or emerges through time? (e.g. antipathy between co-wives, partner infidelity)
• What is cumulative through time? (resulting from successive experiences across a span of time, e.g. chronic illness)
• What decreases or ceases through time? (e.g. romance, marital support)
• What remains constant or consistent through time? (recurring and regularised features of everyday life, e.g. the support from a parent or sibling)
• What kinds of surges, epiphanies or turning points occur through time? (experiences of a magnitude to significantly alter perceptions or life course, e.g. infertility, the death of a child, new love)
• What is idiosyncratic through time? (inconsistencies, unpredictable events, e.g. moving house, death of a sibling and distribution of their children, market closure)
• What is missing through time? (e.g. family support, an independent income)

We manually completed two A3-sized matrices for each of the 16 women, the first covering the year after the ‘near miss’, the second covering the period up to and including the new wave of interviews three to four years later. We noted key attributes such as education, religion, ethnicity, number of children, living conditions and details about the obstetric complication, and listed preliminary analytical assertions. This process facilitated review of the data categorically and comparatively across time. In the final stage, SM and KS analysed the women's experiences for commonality and difference, and grouped and reordered the findings into the set of themes. All names of interviewees referred to below are pseudonyms.

## Findings and discussion

### Structural inequities

Women's accounts of events and relationships revealed common underlying features in the ebbs and flows in the four years following their obstetric emergencies. Structural inequities took their toll and households had to make difficult choices in the face of many demands and difficulties. External factors ranging from seasonality, temporary market closures, to influxes of Chinese goods and the vagaries of world cotton-trading market, put severe pressure on poor households. The weakness of the health system, the high costs of healthcare for obstetric complications and subsequent ailments severely compounded their struggle. There were constant reminders of the fragility of life – the deaths of young adults were followed by the redistribution of their children among remaining siblings, many interviewees had suffered repeated stillbirths and late miscarriages, and all the interviewees had themselves narrowly missed death.

Gender is an essential principle of social organisation (Obrist et al., 2010), and social capital derived from ethnicity and from gender roles had provided certain key endowments to women in these communities. One example was the Mossi male's customary obligation to care for a woman carrying his child and to take responsibility for associated healthcare costs (regardless of formal marriage). As demonstrated in the stories that follow, women's decisions and trajectories were also defined and curtailed by the customary beliefs and obligations relating to family roles and by the everyday social production of gender in social interactions (West & Zimmerman, 1987). These beliefs and obligations included the high value placed on fertility, the importance of boy children in rural families for farming and for provision in old age; the retention of children in the father's household if a couple separated; the stigma of being an unsupported unmarried mother; and the pressures to conform within an unhappy marriage, especially if it had been arranged by parents or elders.

### The turning point of obstetric ‘near miss’

The back-story to each of the sixteen accounts was that of a survival in the face of an obstetric emergency achieved by the mobilisation of social and economic capitals via household, extended family or community networks, which enabled women to access emergency healthcare. The physical, financial and social consequences of the obstetric ‘near miss’ events were highly visible in the ethnographic data from the first year of follow-up (Storeng et al., 2008, 2010). For almost all therefore, the ‘near-miss’ event was a ‘turning point’ that significantly altered their perceptions or life course. Physiological after-effects of the life-threatening events were reflected in a reduction in bodily capital, including greater fatigue, headaches, backache and inability to pursue hard physical work with the same energy as previously (Storeng et al., 2012). The impact was particularly acute for the many households that depended on agricultural labour and heavy domestic activities. The speed and extent of recovery depended on each woman's general state of health, and on the extent of their access to cash for treatment of ongoing illnesses such as anaemia or hypertension, as well as their access to social and material support.

### Descents – resources diminished or denied

The health crisis nudged some women with slim social capital, already buffeted by poverty and illness, further in a downward descent from which recovery was extremely hard. Farida's story, for instance, was one of incremental descent into destitution. Her first husband had died, almost certainly of AIDS, and she too had contracted HIV. Farida had accepted an arranged remarriage, the traditional form of social protection for a woman in her position. As noted elsewhere, the social capital that provides particular gender-specific advantages to women themselves may also simultaneously include dis-benefits specific to those very women (Silvey & Elmhurst, 2003). Over time, Farida's entry into a new household brought new problems. Rivalry with her new co-wife was compounded by the costly medical care of her ‘near-miss’ and her ongoing ill-health, which made her unable to contribute as expected:

*I am accused of laziness, of not wanting to do the agricultural work… Do snide remarks and complaining ever stop between co-wives? No, that's the custom.*

Farida's loss of bodily capital as her illness progressed affected her capacity to call upon social obligations of reciprocity from her co-wives. This further isolated her and compounded her misery. For a while she was able to access antiretroviral treatment thanks to the formal social networks of a healthcare project, and she even succeeded in achieving a much desired pregnancy, but then her supply of drugs ceased. Farida became increasingly ill, poor, disappointed and lonely over the ensuing years. She became unable to do physical work and her husband eventually abandoned her, taking another new and healthier wife.

There were other unrelentingly tragic situations, and these emphasise how inequities can become normalised and reproduced in everyday social relations with casual cruelty. Assetou, an uneducated girl from a rural village worked as a maid for relatives in town. In her case, the social network of the extended family failed to provide social support, but instead served the interests of more powerful members by supplying her bodily capital to them as cheap domestic labour. The aunt and grandmother for whom she worked were both physically abusive to her. At fourteen, Assetou became pregnant, reportedly after a forced sexual encounter with a young male neighbour. Pregnancy brought stigma and further exacerbated her powerlessness in her household. When she developed severe pregnancy-induced hypertension, Assetou experienced a brief respite due to the customary obligations of paternal responsibility.

*My child's father paid for everything. He has the prescriptions, I don't know how much he paid, he never told me.*

Thanks to this, both Assetou and her baby survived. But after the birth, the paternal family's sense of obligation to her waned. With little rest after hospitalisation, Assetou returned to her domestic labours and to work in the family kiosk. Still a child herself, she struggled to care for her baby with little support or interest. At two years old her daughter fell ill.

*They told me to go to take little Aicha to my relatives, so that they could care for her and she wouldn't die here, with them.*

Initially her daughter seemed to recover with medication from the pharmacy. But them she became sick again.

*The second time she woke up and didn't appear ill, but they said her anus was red and it was ‘kotiguai’. I washed her and went to the market with her. On our return I gave her something to eat and she vomited and so it went on... I asked if she could be taken to an old woman in the neighbourhood so she could look at her. When we got there she was dead in my arms…. the illness wore her out and she died.*

Although customary ties and obligations afforded both Farida and Assetou some temporary protection in a time of difficulty, these proved limited and insufficient to avert further distress. Samira's story indicates how the consequences of economic debt incurred by hospitalisation and the accompanying social debt may spiral on into marital disharmony. Samira was a vendor outside the cotton factory. She had three children and also looked after the children from her husband's previous marriage. She had barely survived the obstetric crisis – she suffered high blood pressure, an emergency caesarean section, then two further operations including a hysterectomy. The hospitalisation was extremely costly, but her situation was notable in the first year for the great care and affection shown by her husband during her recuperation. However, the social consequences of these difficult events became more evident over time. Her husband was now indebted to friends who had lent him money for her care. He spent more and more time with them and away from home, to Samira's dismay and unhappiness. Availing herself of an opportunity to remove herself from the situation, she went to Mali for several months to visit her sick father, only to discover on her return that her husband had taken a new co-wife.

*Once I was there my husband took another wife and decided to divorce me after all the disagreements we have had. …… This marriage was sealed without even asking me... they took advantage of my departure to get married behind my back so that, if I learnt about it while I was over there, I would get upset and refuse to come back home.*

In response to this insult, Samira withdrew sexual relations and her domestic labour. There were increasingly violent marital disputes. She now found herself fighting to retain a position in the household. Samira's reduced bodily capital – due to the emergency hysterectomy she was not longer fertile – gave her fewer options to move on and remarry:

*It's difficult for me in the state that I'm in, to choose to go and live with another man now. I can't have children anymore because my child-bearing intestine has been removed.*

### Resources mustered and deployed

The women in our study had few personal assets and little access to cash. The financial costs of emergency obstetric care and subsequent hospitalisation had been incurred by the male partner or head of household who controlled its principle assets, but women also contributed any small resources at their own disposal, depleting future petty trading reserves. A household also pulled through the financial crisis by mobilisation of their immediate social networks – husband's friends, employers, religious groups and extended families. Women often exploited similar personal social capital in subsequent years in order to regain means to generate small independent incomes. These were used in part to restore or enhance bodily capital and, with this, secure social position and security. This route to recovery was far more open to a woman who had a specific benefactor – an ‘uncle’ or ‘aunt’ from the same ethnic group, siblings with salaried jobs or businesses, generous in-laws, or children of working age – than those did not. In a society where almost everyone trades something, however small, women also drew support from social networks relating to their informal economic activities.

Efforts to protect and deploy bodily capital played a key role in some trajectories. As indicated by Samira's loss of power from a hysterectomy, fertility was a key component in women's bodily capital and, once threatened, had to be proved at all costs. A cumulative desperation to prove their ability to give birth to healthy babies dominated the actions of many of the women during the years following their ‘near miss’ resulting in coping strategies of calculated risk taking, often against medical advice and sometimes against partners' wishes. Kadidia, for example, was ill throughout her second pregnancy and had a late miscarriage at seven months, followed by life-threatening sepsis. Her story was dominated by cumulative animosity and competition with a co-wife who she feared might replace her in her husband's affections.

*We often don't communicate for days, and this is getting more frequent… Day before yesterday, I was ill and incapable to cook. Instead of my co-wife taking her place in the kitchen and me repaying her when I am feeling better, oh no! she stayed there without helping to prepare the breakfast. Our husband had to go out and buy something to eat because there wasn't anything!*

Such refusals to collaborate over the common tasks were common manifestations of the jealousies and power struggles that can erupt in polygynous households. For Kadidia, the loss of her baby and the antipathy with this new and fertile co-wife threatened her felt status in the household to such an extent that she defied her husband's concerns about the risks of another pregnancy. She arranged to undergo fertility treatment, meeting the financial costs for this herself.

Habibata's story exemplified that of many of the young women, in that her bodily capital – her vitality, her proven fertility and her looks – was her principle negotiating asset to secure financial and social advantages in a situation of great difficulty. Habibata had undergone a catastrophically expensive emergency caesarean section for pre-eclampsia in her first pregnancy.

*They said the illness was bad, and to have the operation. My mum was scared as we had nothing, because the young man who had made me pregnant had fled and my mother had nothing.*

Habibata's mother and sister borrowed money for her emergency. The daughter of a muezzin, without formal education and from a very poor family, she was acutely aware of the financial burden she had become to her family. Habibata's solution was to accept an offer from a much older man to become his second wife. To win Habibata's consent, this man promised to care for her and also her family. However, his attention to her child and her parents soon faded and she was deeply unhappy with her situation. He wanted more children but Habibata resisted. She began to braid hair to earn a tiny independent income, enough to secretly purchase contraception. Eventually, deprived of food by her husband, she left him, returning to her now-widowed mother. Despite their obvious poverty, the man demanded financial compensation for economic resources he had ‘wasted’ on the girl.

*He had filled out some papers which he brought with him to say that the expenses which he had paid me during our traditional marriage, all the expenses he'd made for me, all these had to be repaid to him by my mother.*

This demand was firmly rebuffed by Habibata's consistent support, her mother, who promptly demanded a higher sum in reparation for her daughter's eleven months of domestic and sexual labour.

### New turning points and reversals of fortune

By the study's second wave, a remarkable turnaround had taken place in Habibata's life, highlighting the interactions of bodily, social and economic capitals. Still young, fertile and considered beautiful, Habibata had secured a new and happier relationship. It was a love match with a young man from a comfortably-off family, and there was a new baby, which had fortunately been born without problems. Habibata was visibly better nourished and happy: ‘*my spirit has settled*’. Her new partner and his father were building her a house. She was working in the fields, selling mangoes and planning to be an apprentice hairdresser.

The longer view afforded by the two waves of the study reveals that reversals of fortune can occur in either direction, positively when new social capital is acquired over time, and negatively when the mobilisation of social capital at time of crisis incurs subsequent social debts. As illustrated by the turn of events in Habibata's life, women often acquired new social capital through new intimate relationships and marriage. However, they also acquired it in other ways. Solange was in her late thirties when she experienced the ‘near-miss’. Her arranged marriage was failing and her husband was living away. She had two children, but she had lost another four around the time of their birth:

‘*the ones that did not survive, I cannot forget. I think about them all the time, even now, I can never stop*. *You suffer and it's all in vain… pointless suffering after much effort, suffering for nothing.*’

With this pregnancy, she had a uterine rupture, losing her baby and her fertility. Her husband came home only to bury the child. At first, her neighbours lent her essentials, but as Solange became more ‘needy’ and depressed, they began to avoid her and to comment that she was crazy. For income, her two remaining children tried to sell small items in the neighbourhood streets. Eventually, the family was evicted for defaulting on rent. For Solange, a religious network gave her a new opportunity. A pastor helped her to find new accommodation. Her new neighbours were welcoming and supportive:

*If people devote time to me I say they are offering me riches… I tried to take pills to put an end to my life... people stopped me, saying I could not do that.*

Although still fatigued, her connections with social life began to return. A local baker gave her credit so that she could sell baguettes in the neighbourhood; ‘*it is selling the bread* ..*that saves me*’.

### Mobility and the exercise of self-displacement

Our longitudinal analysis allowed a heightened perception of the permeability and fluidity of household membership over time: co-wives came and went, children moved to stay with relatives for schooling, maids came to town from the countryside. Moving around was also a feature of economic activity that required work away in the fields or movement for trading. Men were particularly mobile, often going elsewhere for work, including to neighbouring countries. The extent to which social capital could be mobilised was contingent on the configuration of other social actors who were present at any particular time, a situation reflected in Alima's lament that ‘*all those who support me go away*’. Women's access to economic capital and to social capital could be weakened when elder relatives died or when husbands were absent. Although conversely a husband's absence could allow wives freedom to develop independent strategies of survival, through income generating work or the establishment of new social, or sexual, relationships.

Solange's eviction resulted in a move only five km away, but it brought a new life with new opportunities for support and community. Hawa, on the other hand, actively pursued a serial (and ultimately destructive) ‘moving on’ in order to evade personal difficulties. By the time she was twenty she had traversed five households in four years, had two children in different towns, leaving each behind with their respective father. She had become adept at mustering and deploying the capitals at her disposal – her own bodily capital as a young and attractive woman, and the social capital afforded by the obligations of the extended family and by norms of paternal responsibility. As a teenager, Hawa had come as a refugee to stay with her uncle in a relatively privileged household to pursue her education. At fifteen she got pregnant, concealing this for six months before her dismayed uncle sent her to stay with the boyfriend's family, as was customary. Severe pre-eclampsia precipitated the birth. The paternal family paid for hospital care, Hawa and her baby survived. After a failed attempt to combine schooling with breastfeeding she left her baby with its paternal grandparents, returning to her uncle's house. When disputes with his wife spiralled, she moved on again, to live with another of her uncle's wives in another town. There was a fresh start at a new school, but by eighteen she was pregnant once again, with a teacher from the school. She went to live in a house in his yard, but when his first wife left him, Hawa suddenly found herself responsible for the household, her new baby and his four other children.

*I'm happy to have my baby, As for Mr …, it's not as I hoped when we met. I'm not free yet. There are days when I am doing the cooking and a big wash at the same time, and there is a child trailing after me. It's often very tiring for me on my own. Yes, very tiring… I am not as happy as I would have wished.*

Soon after this, unable to cope, she moved out and returned alone to her relatives' home.

We discovered when re-contacting women for follow-up interviews that they often move elsewhere temporarily. As in Hawa's case, unmarried women in the Mossi tradition normally join the partner's household to be cared for during pregnancy. Another traditional form of displacement for women is to spend time at their own family's homestead to recuperate following a birth. This is a method of contraception and reduces consumption of resources in the marital household at a time when they cannot contribute their labour. But more than this, people who appear powerless used the ability to remove themselves from a given situation as a way of precipitating change. Typically they would temporarily move to another household from which they could exert obligations of shelter and provision. Withdrawal from the marital to the parental home, where love was a constant, was thus a common strategy at times of discord. Women and girls do generally face severe mobility constraints in rural areas of sub-Saharan Africa (Porter, 2011) and they might often seem trapped with very little manoeuvre in their traditional roles. Nevertheless, many could and did exercise personal agency in this manner, whether to rest while avoiding accusations of laziness from co-wives, to dissipate tensions within the household, or to force a decision. Fatimah, for example, used withdrawal to her parental home to persuade her boyfriend to marry her:

*When I had [her baby] I effectively went back there, I spent five months with my family…he had a made a promise he didn't keep. That's why I went to stay with my family.*

Other women used self-displacement as a weapon of negotiation at times of marital conflict or to remind a man of the value of their productive and domestic labour. Indeed women's threats to leave were quite commonplace over the course of relationships. Such strategies are not without risk, however, as in Samira's trip home to Mali, which backfired and resulted in new conflicts and a further loss of power.

## Conclusion

Qualitative longitudinal research places central analytical focus on change and continuity over time (Holland, 2011). This emphasis proved key to understanding how individual women in poor communities attempted to muster and deploy social and bodily capital during dangerous reproductive years.

Saldaña's (2009) matrix approach proved extremely useful in establishing the contours of a large dataset and in approaching it in a systematic manner. As far as we are aware this is the first time that it has been used for data from a low-income country setting. Such prospective QLR can help to extend the reach of healthcare enquiry, illuminating ‘what happens afterwards’, by accompanying individuals on their trajectories of adaptation over time.

Healthcare crises play out over time for the people they afflict and affect, and their consequences can be life-changing. When medical treatment is costly, social norms of obligation and reciprocity within kin networks can, and do, enable some poor women to access essential economic resources for the emergency and beyond. But when research looks out into the beyond of people's lives, we can see all too clearly the ways in which weaknesses in health systems – such as high costs for emergency care – interact with and reinforce existing inequities. The women described in this article were not powerful in broader society. The need to survive within a context of severe economic hardship defined their contributions to the household domestic and agricultural spheres and their expectations from it. A key thread running through the sixteen stories is that social capital in such impoverished environments can rarely be mobilised without social costs or consequence.

The detail of their trajectories supports Bebbington's (2007) suggestion that social capital is most usefully understood as a composite of social networks and the resources controlled within them. Actors within a social field have differential access to capitals, which defines relations of domination, subordination or equivalence (Obrist et al., 2010). Our work confirms the importance of understanding specifics of intra-household relationships and the gendered and intergenerational conflicts and hierarchies within social networks (Mayoux, 2001; Silvey & Elmhurst, 2003) and how these reflect structural inequities that set the terms of engagement (van Dijk, 2011). It is this second element of our analytical focus that helps us to understand how, in conditions of great insecurity, an individual's social capital ebbs and flows over time, resulting in a trajectory of multiple adaptations. It was indeed often ‘successive everyday events’ (Krishna, 2010) that caused women's meagre financial, bodily and social resources to fray away. The sale of petty trading assets to meet healthcare costs meant that women struggled to re-enter independent entrepreneurial activity. When health risks became persistent, these challenged both individuals' and households' capacity to mitigate the negative social and economic consequences of illness. Additions to and absences from households could temporarily bring or deplete essential resources.

Anthropologists such as Chapman (2003) have documented the competition among women for scarce resources, including male support and income, in environments of economic insecurity. We have likewise shown that becoming ‘costly’ by causing an unanticipated financial burden on the household reduces a woman's ability to call on future support within its social network. Capacity to recover their situation therefore may depend on whether a woman can mobilise capital from her own kin or a peer network out-with the marital household. While any notion of resilience as ‘bouncing back’ seems quite ludicrously out of place here, our study shows that women are resourceful with whatever they have, as illustrated in the use of self-displacement to the parental home in times of marital dispute, laying claim to the value of their domestic and agricultural labour through its temporary withdrawal. Moreover, those with youth and vitality on their side can use it to turn their life around. Women's capacity to harness or exploit bodily capital in its various forms to some extent determines their ability to confront and overcome adversities. With this, they further mobilise social capital without incurring excessive debt, or access and accumulate significant new social capital. Bodily capital can be an important means for women to facilitate access to new social capital and economic capital, commonly via establishment of intimate relationships or formal marriage arrangements. Conversely, diminished bodily capital following an obstetric complication can reduce opportunities, and lead to further disadvantage.
